# Exploring hemorrhagic shock as a complication of negative pressure wound therapy in the treatment of lumbar spine infection: a case study and literature review

**DOI:** 10.3389/fmed.2025.1602481

**Published:** 2025-08-14

**Authors:** Ge Zhang, Junlin Pan, Shiyong Wan, Zhengqi Chang

**Affiliations:** ^1^Department of Orthopaedics, The 960th Hospital of PLA, Jinan, China; ^2^Department of Reproductive Medicine, The 960th Hospital of PLA, Jinan, China; ^3^Department of Orthopedics, 961th Hospital of PLA, Qiqihar, China

**Keywords:** negative pressure wound therapy technology, lumbar infectious diseases, Brucella, complications, hemorrhagic shock

## Abstract

Lumbar infectious diseases are serious debilitating conditions, and their treatment poses significant challenges. With the advancement of minimally invasive spinal techniques, negative pressure wound therapy (NPWT) has been widely recognized as an effective method for preventing and treating infected wounds. However, studies specifically focusing on hemorrhagic shock as a complication of NPWT in lumbar spine infections are limited. Here, we present a case of hemorrhagic shock as a postoperative complication of Brucella infection in the lumbar spine, and summarize the management of postoperative complications of lumbar infectious diseases treated with NPWT.

## Introduction

Negative pressure wound therapy (NPWT) is a classic treatment method used to treat various complex wounds, widely applied in surgical fields such as diabetic foot, burns, and spinal infections (1). In 1997, the Argenta (2) team first used negative pressure sponge material to promote wound healing and granulation tissue formation, defining it as NPWT. Its advantages, such as promoting granulation tissue formation, microvascular generation, and reducing tissue edema, have been widely recognized, but its complications have not received enough attention. In 2009, the Food and Drug Administration (FDA) in the United States reported serious complications related to NPWT, with six deaths and 77 cases of severe injury caused by NPWT (3). Boxall et al. (4) analyzed 15 patients who received both NPWT and anticoagulant therapy in 2017 through a literature review. One of these patients experienced a bleeding related death (4). During anticoagulant therapy, the occurrence of bleeding complications during NPWT treatment is mainly considered to be due to the immaturity of microvessels. Our team successfully applied NPWT in the treatment of infectious diseases of the lumbar spine and achieved good clinical results (5). During the treatment of a patient with Brucella lumbar spine infection using NPWT, the patient experienced hemorrhagic shock, but after active treatment, no serious adverse consequences occurred. Here, we report the case in detail.

## Case presentation

**Figure 1:**
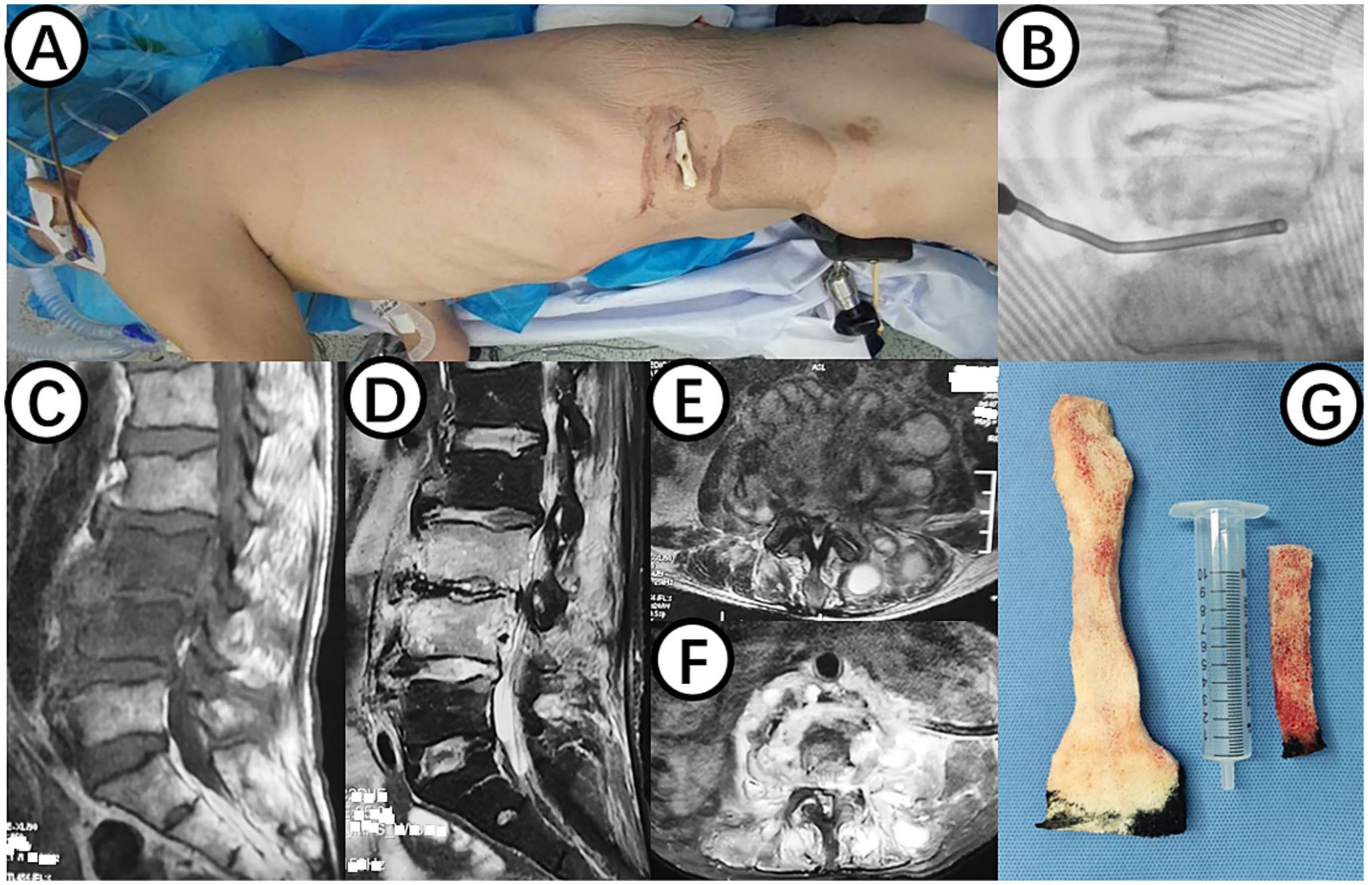
**(A)** Shows the surgical position for changing NPWT. **(B)** Displays intraoperative C-arm fluoroscopy positioning. **(C,E)** Present preoperative MRI scans showing high signal in the L3 and L4 vertebral bodies and intervertebral space, as well as abscess in the intervertebral space, bilateral lumbar muscles, and paraspinal muscles. **(D,F)** Demonstrate postoperative MRI showing decreased inflammation and edema signals in the L3 and L4 vertebral bodies and intervertebral space, with negative pressure sponge and drainage tube in place. **(G)** Depicts the NPWT sponge inside the body being replaced.

A 53-year male presented with lower back pain accompanied by fever for over a month. He was admitted on July 7, 2023, and physical examination revealed severe limitation of lumbar spine flexion and extension, tenderness and percussion pain in the lower lumbar spinous processes and interspinous spaces, with no radiating pain in the lower limbs. Muscle strength was graded as 3/5 in the right quadriceps femoris muscle, 4/5 in the right tibialis anterior, 4/5 in the right triceps surae, and 4/5 in the right ankle dorsiflexors. The straight leg raise test was positive on the right side. MRI findings showed abnormal signals in the L3 and L4 vertebral bodies, multiple abnormal signals in the surrounding lumbar muscles and left posterior soft tissues, as well as posterior slippage of the L3 vertebra and subcutaneous soft tissue edema in the lumbar region (Figures 1C,E). Laboratory tests revealed hemoglobin level of 91 g/L, CRP of 73.69 mg/L, ESR of 80, albumin of 29.6 g/L, globulin of 41.4 g/L, potassium of 3.47 mmol/L, sodium of 132.0 mmol/L, iron of 7.0 μmol/L, and calcium of 2.05 mmol/L. Diagnosis: (1) L3–L4 vertebral osteomyelitis and (2) secondary multiple psoas abscesses.

The patient underwent percutaneous puncture guided by ultrasound upon admission, and on July 8, 2023, he underwent lumbar vertebral infection lesion clearance and drainage surgery under local anesthesia using a percutaneous endoscopic approach. Necrotic tissue was removed, and a tri-cavity drainage tube was placed in the L3/4 intervertebral space. Postoperatively, he received antibiotic therapy for infection control. Brucella agglutination test was positive, and he was given intravenous doxycycline hydrochloride and sulfamethoxazole tablets.

On July 20, 2023, a procedure was performed to clear the lesion in the lumbar spine using an extreme lateral approach. The abdominal external oblique muscle, internal oblique muscle, and transverse abdominal muscle were bluntly dissected to expose the peritoneum perioperatively. The lumbar erector spinae muscle was then exposed, showing swelling and severe inflammation. A purulent abscess was found within the lumbar erector spinae muscle, which was thoroughly drained. The L3/4 vertebral body and intervertebral space were exposed, and necrotic and purulent tissues were completely removed. The wound was thoroughly cleaned, with a small amount of fresh blood oozing from the intervertebral space. A NPWT sponge was placed in the L3/4 intervertebral space (Figures 1A,B), covered with a biological dressing and continuous negative pressure suction, with no obvious air leakage observed.

On the 3rd postoperative day, blood test results showed albumin 28.4 g/L, sodium 132.0 mmol/L, ESR 34, and hemoglobin 119 g/L. A total of 420 mL of fluid was drained in the following 7 days. Due to the patient’s long-term need for intravenous infusion, a peripherally inserted central catheter (PICC) was placed in the right upper limb on July 25, 2023. A Doppler ultrasound on August 3, 2023, revealed a thrombus in the right subclavian vein, for which therapeutic anticoagulation was administered (calcium heparin injection 0.4 mL subcutaneously every 12 h).

**Figure 2:**
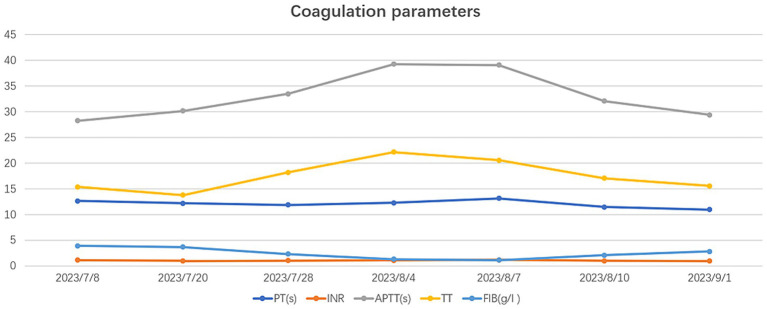
The figure illustrates the changes in five coagulation indicators.

**Figure 3:**
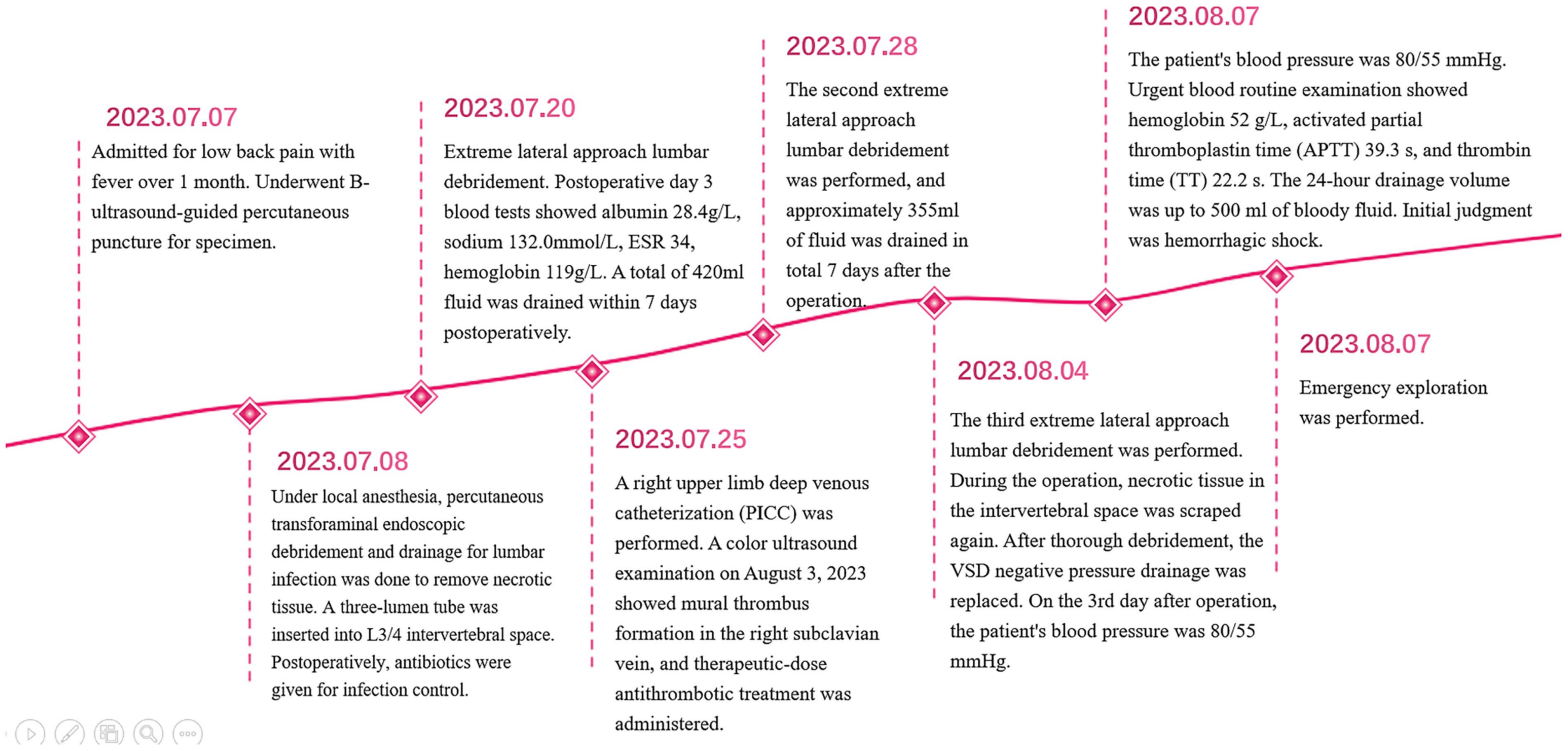
This diagram describes the treatment process according to time points.

**Figure 4:**
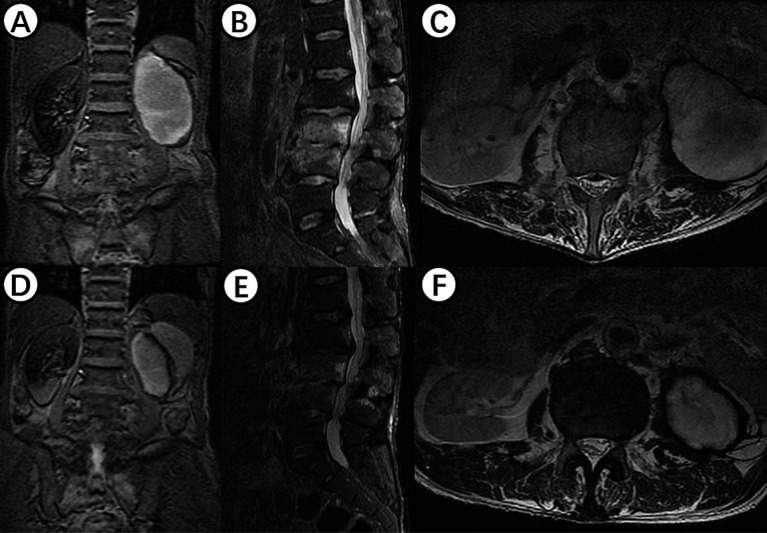
**(A–C)** Shows MRI examination of the lumbar spine in a patient with hemorrhagic shock, revealing an oval-shaped fluid signal at the lower pole of the left kidney, likely due to hematoma at the surgical site. **(D–F)** Displays findings from an MRI examination 3 months postoperatively, showing significant reduction of the hematoma signal, with disappearance of inflammatory signals in the L3 and L4 vertebral bodies and intervertebral space.

On July 28, 2023, the patient underwent the second minimally invasive lateral approach lumbar spine lesion clearance surgery. Approximately 355 mL of fluid was drained postoperatively for 7 days. Prior to the surgery, a follow-up MRI was performed, which showed significant improvement at the site of the lesion (Figures 1D,F). On August 4, 2023, the patient underwent the third minimally invasive lateral approach lumbar spine lesion clearance surgery. During the surgery, necrotic tissue was scraped from the intervertebral space, the wound was thoroughly cleaned, and a NPWT negative pressure drainage was replaced (Figure 1G). On the third day after surgery, the patient’s blood pressure was 80/55 mmHg, and blood tests showed a hemoglobin level of 52 g/L, an activated partial thromboplastin time (Activated Partial Thromboplastin Time) of 39.3 s, and a Thrombin Time (TT) of 22.2 s (The changes in coagulation indicators are detailed in Figure 2). The drainage volume reached 500 mL of bloody fluid in 24 h, indicating hypovolemic shock. Emergency exploration on August 7, 2023, revealed arterial blood oozing from the L3/4 intervertebral space with poor clotting. The coagulation parameters on the same day suggested that the patient was in a peak state of hypocoagulability (see Figure 2). The wound was irrigated with saline, electrocoagulated to stop the bleeding, and packed with gelatin sponge and hemostatic gauze. The wound was satisfactorily closed with a negative pressure drainage tube left in place. The patient received 4 units of red blood cells on the day of surgery. On the first postoperative day, the patient’s albumin level was 27.2 g/L, and hemoglobin level was 48 g/L. The patient received concentrated red blood cells (AB type), fresh frozen plasma (AB type), and cryoprecipitate, as well as furosemide injection and a saline solution with dexamethasone. The patient received 4 units of red blood cells, 600 mL of fresh frozen plasma, and 10 units of cryoprecipitate on the first postoperative day. On the second postoperative day, the hemoglobin level was 60 g/L, and the patient received 2 units of red blood cells, 610 mL of fresh frozen plasma, and 10 units of cryoprecipitate. On the third postoperative day, the blood pressure was 126/87 mmHg, the hemoglobin level was 87 g/L, and the albumin level was 37.7 g/L. The patient was given two units of red blood cells, resulting in a significant improvement in their anemia symptoms. Refer to Figure 3 for a detailed treatment process. MRI review indicates the presence of a hematoma in the surgical area (Figures 4A–C). After four effective debridement surgeries and antibiotic treatment, the lumbar spine infection improved gradually, and the retroperitoneal hematoma was absorbed. Follow-up abdominal MRI scan after 3 months showed an encapsulated fluid signal in the retroperitoneum (Figures 4D–F).

## Discussion

NPWT, as an effective means of assisting in the treatment of complex wounds, has shown increasing effectiveness in the management of infectious diseases of the lumbar spine. Previous studies have found that NPWT has the following mechanisms of action in treating bone and soft tissue infections: stabilizing the wound environment, reducing wound edema, lowering internal bacterial load, promoting granulation tissue formation, enhancing blood flow perfusion, and strengthening immune function (6). We have successfully used NPWT to treat both primary spinal infections and postoperative spinal incision infections, achieving significant clinical outcomes (5).

With the widespread application of NPWT, the incidence of its complications is increasing. Numerous studies have shown that NPWT can lead to various complications in the fields of cardiac surgery, abdominal surgery, and pressure ulcer debridement (7–14) (Table 1). In 2011, the FDA issued a report on the “latest developments in serious complications related to NPWT systems,” which reported six deaths, with three deaths related to bleeding (15). Although the FDA did not specify that patients receiving anticoagulant or antiplatelet therapy are contraindicated for NPWT, it listed this as one of the risk factors to consider before starting treatment. Additionally, in 2014, the Canadian Agency for Drugs and Technologies in Health (16) released a review on “NPWT for Managing Diabetic Foot Ulcers: Clinical Effectiveness, Cost-Effectiveness, and Guidelines,” which stated that the use of NPWT can be a high-risk factor for postoperative bleeding. In analyzing the postoperative hemorrhagic shock in this case, the possible reason is that the patient was taking therapeutic doses of anticoagulant medication, leading to abnormal coagulation function, compounded by the effects of NPWT causing prolonged and continuous bleeding.

**Table 1:** Reported complications of negative pressure wound therapy in recent literature.

Author	Number of patients	Age (years)	Operation	Trade name	Negative pressure	Complications	Treatment	Follow-up time (weeks)
Gorgulu (7)	1	33	Abdominoplasty	Vacuum-assisted closure system (Prevena 125, Kinetic Concepts, Inc., San Antonio, TX)	125 mmHg	Epidermal necrosis	Gauze impregnated with chlorhexidine was used as a wound dressing for dressing changes 10 times a day.	12
Caianiello et al. (8)	1	9	Cardiac resection and *in situ* heart transplantation	Vacuum-assisted closure system	75 mmHg	Ascending aortic chylous hemorrhage	Resection of the aortic wall covered with thick granulation tissue around the vesicle and a bovine pericardial patch was performed	72
Omran et al. (9)	1	64	Coronary artery bypass grafting	VAC system Vivano (Paul Hartmann AG, Heidenheim, Germany)	130 mmHg	Broncho-pleural dermal fistula formation	Left-sided open-heart surgery	/
Fankhauser et al. (10)	1	66	Open duodenal fistula repair with partial resection of duodenum and jejunum and one-stage enteral reconstruction	NPWT (Renasys-GO™ system, Smith& Nephew GmbH)	/	Uretero-peritoneal fistula	Topical silver-releasing alginate dressings and periodic debridements	72
Rencuzogullari (11)	1	23	Open wound debridement of the perianal area due to blast injury	Vacuum-assisted closure system	/	Sponge retention leading to perianal abscesses and fistulas	Surgical management of perianal abscesses and fistulas	/
Mazoch and Montgomery (12)	2	30, 54	Sacral and right sciatic pressure sore debridement after paraplegia	Negative pressure wound therapy	/	Chronic wound drainage due to retention of foam dressings for surgical wounds	Surgical debridement	288
Stanger et al. (13)	1	86	Wound infection originating from debridement of chronic lower extremity foot ulcers	Negative pressure wound therapy	/	Tissue necrosis and perforation of the anterior tibial artery	Surgical debridement and vascularization	/
Datta et al. (14)	1	56	Coronary Artery Bypass Grafting	Vacuum-assisted closure system	/	Pseudoaneurysm of right internal thoracic artery	Pseudoaneurysm removed	72

The “/” symbol indicates a lack of information.

The patient developed a complication of subclavian vein wall thrombosis after undergoing a right upper limb deep venous catheterization during the perioperative period. Treatment with anticoagulants led to increased drainage fluid volume, dark red color, and a decrease in blood pressure to 80/55 mmHg with a hemoglobin level of 52 g/L. The bleeding was considered to be strongly associated with the use of anticoagulant medication. The risk of severe vascular injury during extreme lateral interbody fusions (XLIF) surgery is reported to range from 0 to 0.4% (17). Peiró-García et al. (18) reported a case of a patient who underwent a simple XLIF surgery, resulting in a life-threatening retroperitoneal hematoma. They emphasized that a rapid change in vital signs can alert spine surgeons to the possibility of major vascular injury. In 2014, Assina et al. (19) also described a case of a 50-year-old woman who suffered severe vascular injury during L4-L5 XLIF surgery, with the intraoperative vascular injury being fatal. Unlike other cases where significant intraoperative bleeding occurred, this patient had previously undergone two XLIF surgeries without any vascular complications, suggesting a lower correlation between the current vascular injury and the surgical technique. Additionally, the use of NPWT at appropriate pressure levels can help achieve hemostasis. Therefore, it is likely that in this particular case, the patient experienced bleeding due to a combination of anticoagulant therapy and damage to small blood vessels from multiple previous surgeries.

Negative pressure wound therapy can make it challenging to detect signs of bleeding early in the treatment process due to two main factors. Firstly, the sealed dressing can obstruct the wound, causing blood to be absorbed into the dressing or sponge, making external bleeding hard to detect. Secondly, blood can mix with accumulated secretions in the negative pressure drainage bottle, potentially leading to misinterpretation as normal exudate. To prevent this issue, clinicians should carefully choose NPWT for high-risk bleeding wounds, set appropriate negative pressure values, observe the dressing’s appearance and drainage fluid characteristics, monitor vital signs, and use auxiliary monitoring techniques like blood oxygen monitoring. Extra caution should be exercised with patients on anticoagulant therapy by assessing and monitoring their coagulation function.

## Conclusion

We reported a case of NPWT treatment for lumbar spine infection, which resulted in postoperative hemorrhagic shock complications. By analyzing the cause of bleeding, it was recognized that the use of anticoagulants in therapeutic doses is a relative contraindication for NPWT. Clinically, the coagulation function of patients should be strictly grasped, developing an individualized risk - benefit assessment, which has positive significance for standardizing the use of NPWT.

## Data Availability

The original contributions presented in the study are included in the article/supplementary material, further inquiries can be directed to the corresponding author.

## References

[1] Normandin S Safran T Winocour S Chu CK Vorstenbosch J Murphy AM. Negative pressure wound therapy: mechanism of action and clinical applications. Semin Plast Surg. (2021) 35:164–70. doi: 10.1055/s-0041-1731792, PMID: 34526864 PMC8432996

[2] Argenta LC Morykwas MJ. Vacuum-assisted closure: a new method for wound control and treatment: clinical experience. Ann Plast Surg. (1997) 38:563–76. discussion 577 9188971

[3] Shuren JE. FDA preliminary public health notification*: serious complications associated with negative pressure wound therapy systems In: FDA medical device public health notifications, vol. 1. The United States. Silver Spring, Maryland: FDA Medical Device Public Health Notifications (2009)

[4] Boxall SL Carville K Leslie GD Jansen SJ. Treatment of anticoagulated patients with negative pressure wound therapy. Int Wound J. (2017) 14:950–4. doi: 10.1111/iwj.12737, PMID: 28294534 PMC7949645

[5] Xing W Yang Y Bai Y Yu X Chang Z. A comparison of negative pressure and conventional therapy in spine infections: a single-center retrospective study. J Pers Med. (2023) 13:162. doi: 10.3390/jpm13020162, PMID: 36836397 PMC9965435

[6] Xing H Meng QF Chang ZQ. Mechanism of negative pressure wound therapy in the auxiliary treatment of bone and soft tissue. Chin J Tissue Eng Res. (2024) 28:621–6. doi: 10.12307/2023.959

[7] Gorgulu T. A complication of management of closed incision with negative-pressure wound therapy. Aesthet Surg J. (2015) 35:NP113–5. doi: 10.1093/asj/sju120 26026135

[8] Caianiello G Petraio A Ursomando F Pepino P Cotrufo M De Feo M. Aortic erosion during negative pressure therapy in a pediatric heart transplant recipient. Ann Thorac Surg. (2011) 92:1879–80. doi: 10.1016/j.athoracsur.2011.04.092, PMID: 22051284

[9] Omran N Habal P Mandak J Chek JL. Broncho-pleural fistula following vacuum-assisted closure therapy. J Card Surg. (2013) 28:397–8. doi: 10.1111/jocs.12126, PMID: 23711339

[10] Fankhauser CD Hermanns T Hasse B Rancic Z. Excessive wound fluid discharge during retroperitoneal negative pressure wound therapy. Ann Vasc Surg. (2017) 43:314.e1–3. doi: 10.1016/j.avsg.2017.02.017 28479439

[11] Rencuzogullari A. Retention of vacuum-assisted closure device sponge leading to a perianal abscess and fistula. Int Wound J. (2015) 12:739–40. doi: 10.1111/iwj.12200, PMID: 24612666 PMC7950691

[12] Mazoch M Montgomery C. Retained wound vacuum foam in non-healing wounds: a real possibility. J Wound Care. (2015) 24:S18–20. doi: 10.12968/jowc.2015.24.Sup6.S18 26075511

[13] Stanger KM Albert F Kneser U Bogdan C Horch RE. Management of chronic osteomyelitis of the tibia with life-threatening complications under negative pressure wound therapy and isolation of *Helcococcus kunzii*. Int Wound J. (2015) 12:443–6. doi: 10.1111/iwj.12133, PMID: 23855685 PMC7950519

[14] Datta S Manoly I Karangelis D Hasan R. Pseudoaneurysm of the right internal mammary artery post vacuum-assisted closure therapy: a rare complication and literature review. Ann Vasc Surg. (2016) 31:207.e1–3. doi: 10.1016/j.avsg.2015.08.010 26597235

[15] Food and Drug Administration. UPDATE on serious complications associated with negative pressure wound therapy systems: FDA safety communication. Silver Spring, MD: US Department of Health and Human Services (2014).

[16] Zhang G Pan J Wan S Chang Z. Negative pressure wound therapy for managing diabetic foot ulcers: a review of the clinical effectiveness, cost-effectiveness, and guidelines. Ottawa, ON: Canadian Agency for Drugs and Technologies in Health (2014). 25411671

[17] Epstein NE. Incidence of major vascular injuries with extreme lateral interbody fusion (XLIF). Surg Neurol Int. (2020) 11:70. doi: 10.25259/SNI_113_2020, PMID: 32363065 PMC7193196

[18] Peiró-García A Domínguez-Esteban I Alía-Benítez J. Retroperitoneal hematoma after using the extreme lateral interbody fusion (XLIF) approach: presentation of a case and a review of the literature. Rev Esp Cir Ortop Traumatol. (2016) 60:330–4. doi: 10.1016/j.recot.2014.12.006, English, Spanish 25703640

[19] Assina R Majmundar NJ Herschman Y Heary RF. First report of major vascular injury due to lateral transpsoas approach leading to fatality. J Neurosurg Spine. (2014) 21:794–8. doi: 10.3171/2014.7.SPINE131146, PMID: 25192374

